# Changes in each retinal layer and ellipsoid zone recovery after full-thickness macular hole surgery

**DOI:** 10.1038/s41598-021-90955-4

**Published:** 2021-05-31

**Authors:** Min-Woo Lee, Tae-Yeon Kim, Yong-Yeon Song, Seung-Kook Baek, Young-Hoon Lee

**Affiliations:** Department of Ophthalmology, Konyang University College of Medicine, Konyang University Hospital, #158 Gwanjeodong-ro, Seo-gu, Daejeon, 35365 Republic of Korea

**Keywords:** Diseases, Medical research

## Abstract

To analyze the changes in each retinal layer and the recovery of the ellipsoid zone (EZ) after full-thickness macular hole (FTMH) surgery. Patients who underwent surgery for FTMH were included. Spectral-domain optical coherence tomography (SD-OCT) was performed preoperatively and postoperatively at 1, 3, 6, 9, and 12 months. A total of 32 eyes were enrolled. Ganglion cell layer, inner plexiform layer, and inner nuclear layer showed significant reductions over time after surgery (P = 0.020, P = 0.001, and P = 0.001, respectively), but were significantly thicker than those of fellow eyes at 12 months postoperatively. The average recovery duration of the external limiting membrane (ELM), outer nuclear layer (ONL), and EZ was 1.5, 2.1, and 6.1 months, respectively. Baseline best-corrected visual acuity (BCVA) (P = 0.003), minimum linear diameter (MLD) (P = 0.025), recovery of EZ (P = 0.008), and IRL thickness (P < 0.001) were significant factors associated with changes in the BCVA. Additionally, axial length (P < 0.001), MLD (P = 0.020), and IRL thickness (P = 0.001) showed significant results associated with EZ recovery. The IRL gradually became thinner after FTMH surgery but was still thicker than that of the fellow eye at 12 months postoperatively. The recovery of ELM and ONL may be a prerequisite for the EZ recovery. The BCVA change was affected by baseline BCVA, MLD, recovery of EZ, and IRL thickness. Additionally, axial length, MLD, and IRL thickness were significantly associated with EZ recovery.

## Introduction

The standard treatment for full-thickness macular hole (FTMH) is pars plana vitrectomy (PPV) with internal limiting membrane (ILM) peel and intravitreal gas tamponade, of which anatomic success rates were reported as high as 85–100%^1–3^. Surgical techniques have since been developed to improve the anatomic success rates of large macular holes, such as inverted ILM flap techniques and autologous retinal transplantation^4,5^. However, a sealed hole does not guarantee the great improvement in visual acuity with this high surgical success rate. Thus, many studies have attempted to identify the factors affecting vision after treatment of a FTMH.

Modi et al.^6^ reported that a larger ILM peel area is associated with significant alteration in inner retinal architecture, causing the adverse functional outcomes after FTMH surgery. Other studies have also determined that minimizing the structural alterations of the inner retina would improve visual outcomes in patients undergoing FTMH surgery^7,8^. Therefore, the inner retinal layer (IRL) would be one of the important factors associated with the visual outcomes after FTMH surgery. However, few studies have reported the longitudinal changes in the IRL or its relationship with changes in visual acuity after FTMH surgery.

Christensen et al.^9^ reported that structural recovery in the form of photoreceptor layer discontinuity is associated with poorer visual acuity after treatment of FTMH. Bottoni et al.^10^ also reported recovery of the external limiting membrane (ELM) and ellipsoid zone (EZ) is important for the improvement of visual acuity after FTMH surgery. They also insisted that the ELM is the first structure to recover and gradual recovery of the EZ occurs in the presence of an intact outer nuclear layer (ONL). Thus, it is crucial to observe the recovery of the EZ after FTMH surgery.

In this study, we analyzed the process of the changes in each retinal layer and the foveal recovery of the ELM, ONL, and EZ after the anatomic success of FTMH surgery. Additionally, we tried to identify the factors affecting changes in visual acuity and those associated with EZ recovery.

## Methods

### Patients

This study included patients with idiopathic FTMH who were enrolled in the “Investigating changes in retinal thickness after vitrectomy” study, an ongoing prospective investigation at the Konyang University College of Medicine. This prospective observational study adhered to the tenets of the Declaration of Helsinki and was approved by the Institutional Review Board of Konyang University Hospital, Daejeon, Korea, and informed consent was obtained from all of the patients. We obtained detailed histories and best-corrected visual acuity (BCVA), intraocular pressure, spherical equivalent, and axial length^11^. All patients underwent 25-gauge PPV with ILM peeling using indocyanine green, and fluid-gas exchange using C_3_F_8_ or SF_6_ with face down posturing. When combined with cataract surgery, phacoemulsification was performed before vitrectomy, and the combined surgery was performed in patients with grade 2 or higher lens opacity classification [LOC III]. The International Vitreomacular Traction Study Group Classification System method was used to measure the size of the macular hole. The minimum linear diameter (MLD) and base diameter were measured using software caliper^12^. Patients with failure to sealing the macular hole after surgery or complication after surgery such as macular edema, histories of any kind of ophthalmic diseases other than FTMH, intraocular surgery except cataract extraction were excluded. Patients for which the ILM flap technique was used were also excluded for the homogeneity of subjects.

### Optical coherence tomography measurements

Two independent observers (M.W.L. and T.Y.K.) performed detailed analyses of optical coherence tomography (OCT) images. Spectral-domain OCT (Spectralis; Heidelberg Engineering, Heidelberg, Germany) was performed before surgery and at 1, 3, 6, 9, and 12 months after surgery, using the AutoRescan mode. The thickness of the segmented subfoveal retinal layer, the central circle of the Early Treatment of Diabetic Retinopathy Study, was measured automatically using the HRA/Spectralis Viewing Module (ver.6.9.5.0). Manual adjustment was performed when an obvious segmentation error was found. The IRL was defined as the nerve fiber layer (NFL) + ganglion cell layer (GCL) + inner plexiform layer (IPL) + inner nuclear layer (INL). The outer retinal layer (ORL) was defined as the photoreceptor layer (PRL) + retinal pigment epithelium (RPE). Images showing a motion artifact, involuntary saccade, obvious decentration, misalignment, or algorithm segmentation failure were excluded.

### Statistical analyses

Each retinal layer in vitrectomized eyes and fellow eyes were compared using a paired t-test. Repeated-measure analysis of variance was used to identify the change of each retinal layer over time. Univariate and multivariate generalized linear mixed models were used to determine the factors associated with BCVA changes over time. Univariate and multivariate generalized estimating equations were performed to identify factors affecting EZ recovery. Statistical analyses were performed using SPSS software (version 18.0; IBM Corp., Armonk, NY, USA).

## Results

### Demographics

The study initially enrolled 38 eyes in which PPV was performed for a FTMH. Of these, 6 eyes were excluded from the study: 1 eye due to failure to seal the hole after the initial PPV, 1 eye due to the occurrence of macular edema during the follow-up period, and 4 eyes as a result of segmentation error that could not be managed by manual adjustment, resulting in 32 enrolled eyes. The mean age was 64.1 ± 9.6 years, the mean axial length was 24.4 ± 1.7 mm, and baseline BCVA was 0.98 ± 0.42 (Table 1). Three eyes (9.3%) showed FTMH with stage 2, 14 eyes (43.8%) with stage 3, and 15 eyes (46.9%) with stage 4. The average MLD was 391.4 ± 165.8 μm, and the average base diameter was 822.1 ± 211.6 μm.

**Table 1:** Demographics.

Number of case	32
Age (years, mean ± SD)	64.1 ± 9.6
Sex (men, %)	9 (31%)
Laterality (right, %)	17 (58.6%)
Diabetes (n, %)	5 (17.2%)
Hypertension (n, %)	11 (37.9%)
Baseline intraocular pressure (mmHg, mean ± SD)	14.0 ± 3.5
Spherical equivalent (diopter, mean ± SD)	-1.10 ± 2.20
Axial length (mm, mean ± SD)	24.4 ± 1.7
Baseline BCVA (logMAR, mean ± SD)	0.98 ± 0.42
Minimum linear diameter (μm, mean ± SD)	391.4 ± 165.8
Base diameter (μm, mean ± SD)	822.1 ± 211.6

*SD* standard deviation, *BCVA* best-corrected visual acuity.

### Changes in BCVA and each retinal layer thickness after vitrectomy

**Figure 1:**
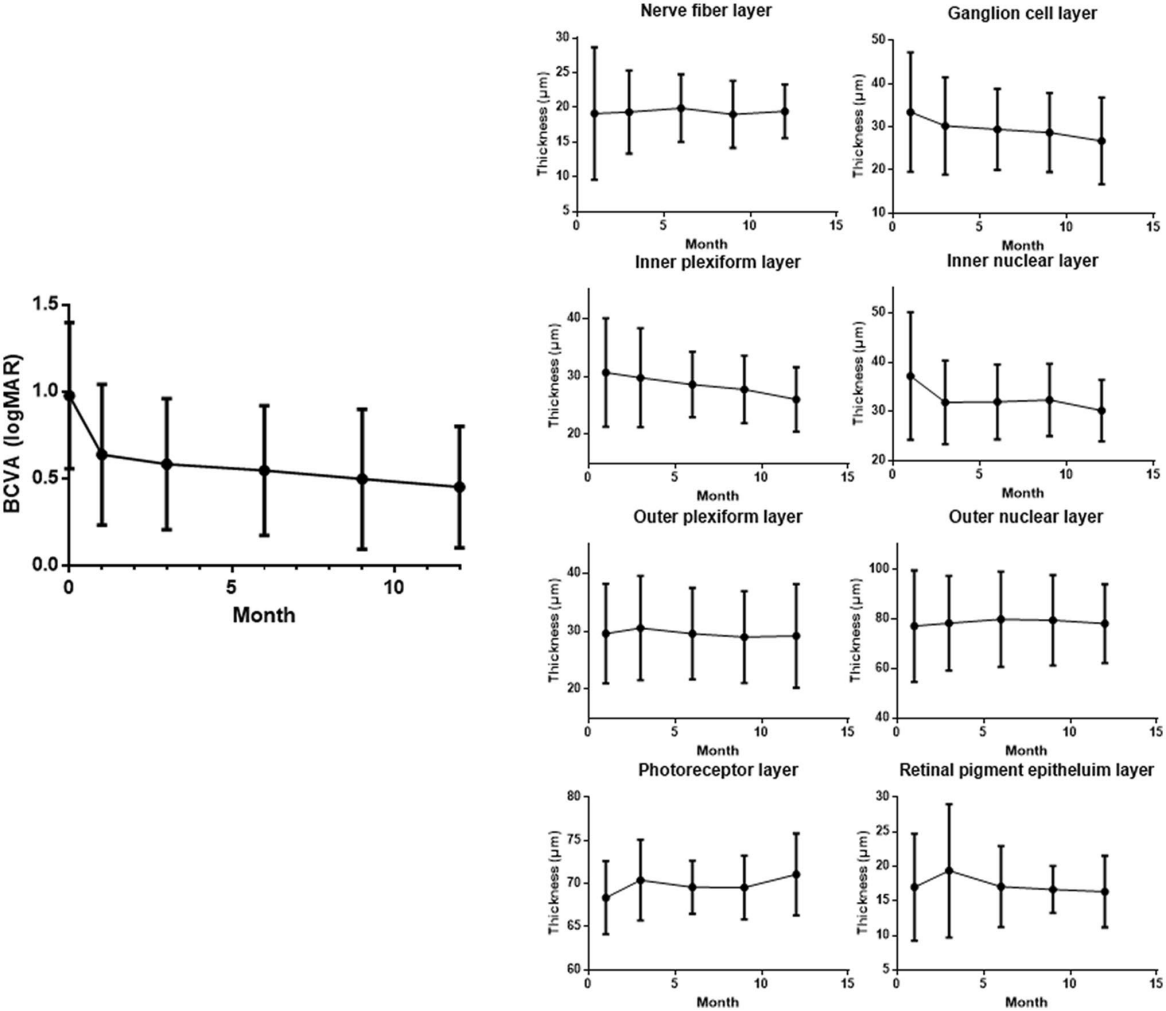
Best-corrected visual acuity (BCVA) and each retinal layer thickness at each visit after full-thickness macular hole surgery. Ganglion cell layer, inner plexiform layer, inner nuclear layer, and photoreceptor layer showed significant changes over time (P = 0.020, P = 0.001, P = 0.011, and P = 0.011, respectively).

The greatest improvement in BCVA was observed in the first month after surgery, with steady improvement over time (Fig. 1, Table 2). The mean final BCVA was 0.45 ± 0.35. GCL, IPL, and INL showed a reduction over time after surgery, and they were statistically significant (P = 0.020, P = 0.001, and P = 0.011, respectively). Whereas, the photoreceptor layer thickness showed a significant increase over time (P = 0.011). The other retinal layers did not exhibit a consistent direction with respect to a change in thickness. Each layer in IRL of vitrectomized eyes was significantly thicker than those of fellow eyes at 12 months after surgery (Table 3).

**Table 2:** The minimum linear diameter of macular hole and visual acuity for each patient.

	MLD	Baseline BCVA	1 month BCVA	3 months BCVA	6 months BCVA	9 months BCVA	12 months BCVA
Patient 1	222	0.90	0.55	0.55	0.55	0.30	0.40
Patient 2	388	0.90	0.90	0.90	0.90	0.90	0.40
Patient 3	308	0.70	0.55	0.70	0.40	0.55	0.70
Patient 4	658	1.70	0.55	0.70	0.55	0.55	0.70
Patient 5	329	0.70	0.17	0.05	0.05	0.05	0.05
Patient 6	508	1.00	1.00	0.90	0.90	0.90	0.70
Patient 7	623	1.70	1.70	0.90	0.90	0.90	0.90
Patient 8	421	1.00	0.40	0.70	0.55	0.40	0.30
Patient 9	425	1.00	0.70	0.90	0.55	0.90	0.70
Patient 10	758	1.70	1.70	1.70	1.70	1.70	1.30
Patient 11	434	1.70	0.70	0.70	0.70	0.70	0.70
Patient 12	370	0.90	0.55	0.55	0.55	0.52	0.40
Patient 13	606	1.00	0.70	0.55	0.40	0.40	0.40
Patient 14	350	1.00	0.90	0.90	1.00	0.17	0.17
Patient 15	145	0.22	0.10	0.00	0.00	0.00	0.00
Patient 16	441	1.00	0.90	0.90	0.70	0.55	0.55
Patient 17	294	1.00	0.22	0.17	0.17	0.17	0.17
Patient 18	391	1.30	1.30	1.30	0.70	0.55	0.55
Patient 19	395	1.00	0.70	0.90	0.70	0.70	0.70
Patient 20	469	1.70	0.90	0.70	0.55	0.55	0.40
Patient 21	102	0.90	0.70	0.30	0.30	0.30	0.30
Patient 22	391	1.00	0.55	0.55	0.52	0.40	0.40
Patient 23	392	1.00	0.70	0.55	0.55	0.52	0.40
Patient 24	55	0.30	0.10	0.10	0.00	0.00	0.00
Patient 25	363	0.70	0.17	0.10	0.22	0.05	0.00
Patient 26	466	1.00	0.90	0.70	0.90	0.70	0.70
Patient 27	312	0.55	0.52	0.55	0.55	0.40	0.40
Patient 28	505	1.00	0.90	0.90	1.00	0.90	0.90
Patient 29	382	0.55	0.22	0.05	0.17	0.17	0.17
Patient 30	189	0.22	0.05	0.10	0.10	0.00	0.00
Patient 31	593	1.00	0.70	0.40	0.40	0.30	0.30
Patient 32	240	1.30	0.70	0.55	0.40	0.30	0.30

*MLD* minimum linear diameter, *BCVA* best-corrected visual acuity.

**Table 3:** Each retinal layer thickness in vitrectomized eyes for full-thickness macular hole and fellow eyes at 12 months after surgery.

	Vitrectomized eye	Fellow eye	P value
SFT	302.6 ± 36.1	264.4 ± 30.7	** < 0.001**
NFL	19.3 ± 4.0	12.3 ± 5.2	** < 0.001**
GCL	27.8 ± 10.8	15.8 ± 10.2	** < 0.001**
IPL	28.3 ± 7.2	19.8 ± 6.0	** < 0.001**
INL	32.7 ± 9.2	21.6 ± 9.7	** < 0.001**
OPL	29.0 ± 9.1	26.4 ± 10.5	0.277
ONL	79.0 ± 15.6	80.5 ± 12.6	0.626
PRL	71.2 ± 4.8	71.9 ± 4.4	0.379
RPE	16.3 ± 5.2	16.3 ± 3.4	0.999

All values are expressed as the mean ± standard deviation (μm). Values in boldface are statistically significant ( P < 0.050).

*SFT* subfoveal thickness, *NFL* nerve fiber layer, *GCL* ganglion cell layer, *IPL* inner plexiform layer, *INL* inner nuclear layer, *OPL* outer plexiform layer, *ONL* outer nuclear layer, *PRL* photoreceptor layer, *RPE* retinal pigment epithelium.

### Factors associated with changes in the BCVA

In univariate analyses, sex (P = 0.046), baseline BCVA (P < 0.001), follow-up duration after surgery (P < 0.001), MLD (P < 0.001), base diameter (P = 0.012), recovery of EZ (P < 0.001), SFT (P < 0.001), and IRL thickness (P < 0.001) showed significant results associated with changes in the BCVA (Table 4). We performed 2 multivariate models to avoid errors caused by multicollinearity with regard to the SFT and IRL. In model 1, baseline BCVA (P = 0.003), follow-up duration (P = 0.034), MLD (P = 0.025), recovery of EZ (P = 0.008), and IRL thickness (P < 0.001) were significant factors. Model 2 showed results similar to those of model 1, with SFT identified as one of the significant factors (P < 0.001).

**Table 4:** Univariate and multivariate linear mixed model determination of factors associated with changes in the best-corrected visual acuity (BCVA).

	Univariate		Multivariate (model 1)		Multivariate (model 2)	
	Estimate (95% CI)	P value	Estimate (95% CI)	P value	Estimate (95% CI)	P value
Age	− 0.006 (− 0.021–0.010)	0.461				
Sex	− 0.293 (− 0.579 to − 0.006)	**0.046**	0.044 (− 0.139–0.228)	0.627	0.023 (− 0.157–0.213)	0.762
Diabetes	0.051 (− 0.327–0.429)	0.783				
Hypertension	− 0.079 (− 0.372–0.215)	0.587				
Axial length	0.001 (− 0.001–0.002)	0.667				
Combined surgery	− 0.144 (− 0.449–0.160)	0.340				
Baseline BCVA	0.701 (0.486–0.915)	** < 0.001**	0.442 (0.154–0.669)	**0.003**	0.424 (0.165–0.683)	**0.002**
F/U duration after surgery	− 0.033 (− 0.045 to − 0.021)	** < 0.001**	− 0.128 (− 0.025 to − 0.001)	**0.034**	− 0.014 (− 0.026 to − 0.002)	**0.025**
Stage of MH	0.102 (− 0.124–0.326)	0.364				
MLD	0.002 (0.001–0.002)	** < 0.001**	0.001 (< 0.001–0.002)	**0.025**	0.001 (< 0.001–0.002)	**0.034**
Base diameter	0.001 (< 0.001–0.001)	**0.012**	< 0.001 (− 0.001–0.001)	0.954	< 0.001 (− 0.001–0.001)	0.810
Recovery of ELM	0.018 (− 0.143–0.179)	0.824				
Recovery of ONL	0.088 (− 0.023–0.199)	0.121				
Recovery of EZ	0.240 (0.142–0.338)	** < 0.001**	0.122 (0.032–0.213)	**0.008**	0.121 (0.028–0.214)	**0.011**
Subfoveal thickness	0.002 (0.002–0.003)	** < 0.001**			0.002 (0.001–0.003)	** < 0.001**
IRL thickness	0.003 (0.002–0.003)	** < 0.001**	0.002 (0.002–0.003)	** < 0.001**		
ORL thickness	< 0.001 (− 0.003–0.003)	0.982				
PRL thickness	− 0.015 (− 0.023 to − 0.006)	**0.001**	− 0.001 (− 0.013–0.001)	0.128	− 0.001 (− 0.015–< 0.001)	0.061

Values in boldface are statistically significant (P < 0.050).

*F/U* follow-up, *MH* macular hole, *MLD* minimum linear diameter, *ELM* external limiting membrane, *ONL* outer nuclear layer, *EZ* ellipsoid zone, *IRL* inner retinal layer, *ORL* outer retinal layer, *PRL* photoreceptor layer.

### Recovery process of EZ and the associated factors

**Figure 2:**
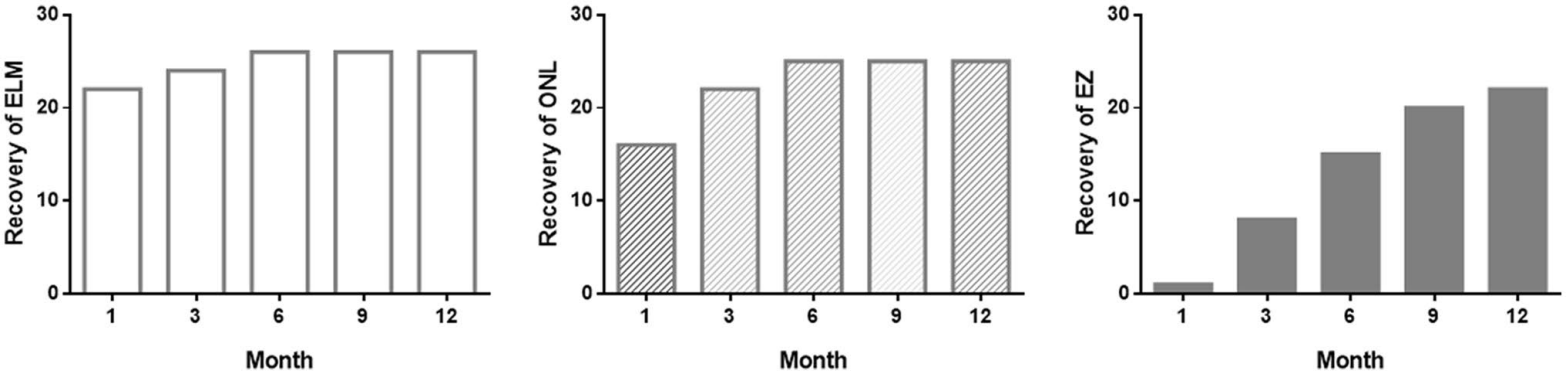
Recovery of the external limiting membrane (ELM), outer nuclear layer (ONL), and ellipsoid zone during follow-up. Cases with an intact ONL had an intact ELM at each visit, and cases with an intact EZ had an intact ONL.

**Figure 3:**
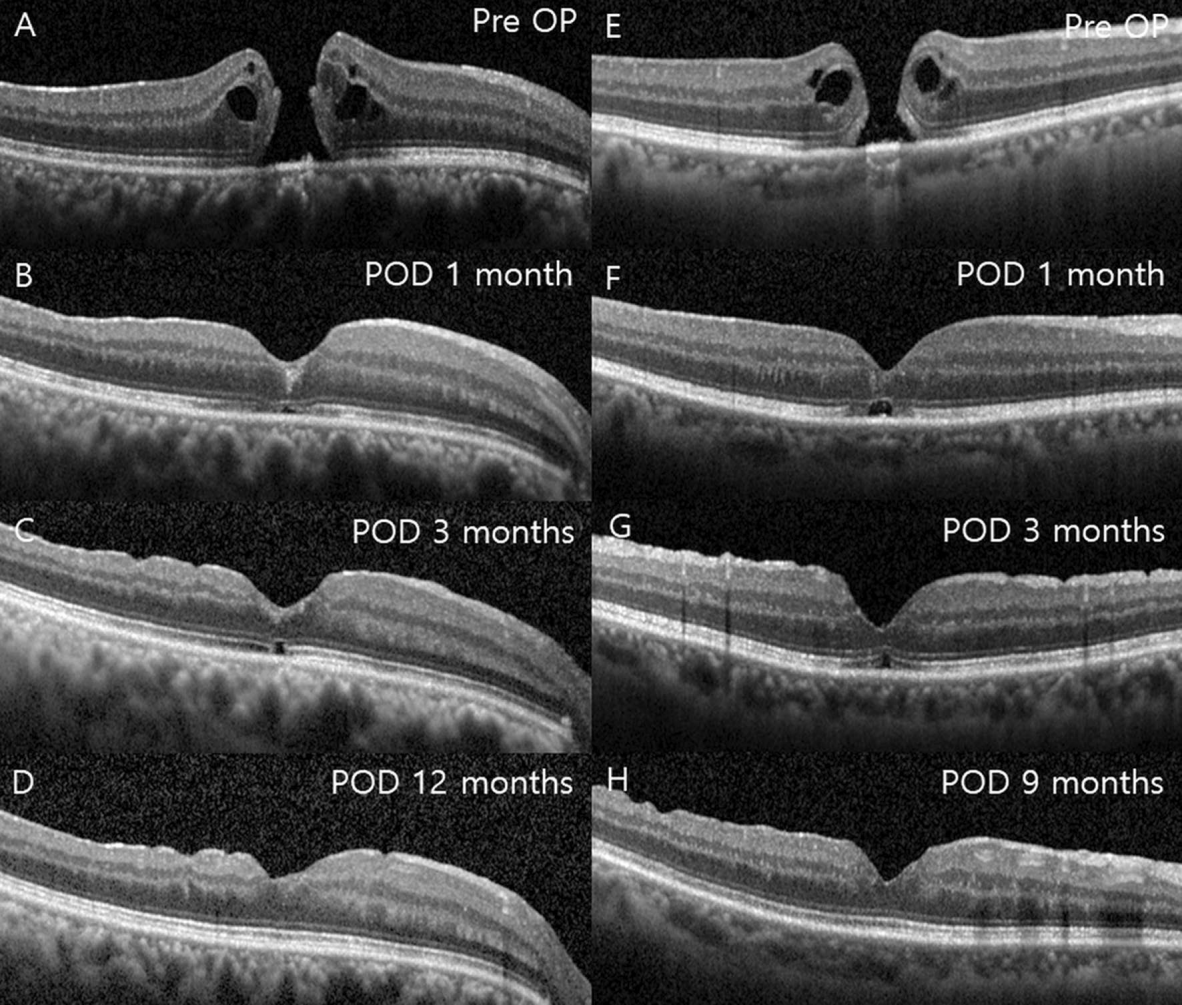
Two cases showing changes in the external limiting membrane (ELM), outer nuclear layer (ONL), and ellipsoid zone (EZ) during follow-up. The first case recovered its EZ at 12 months (**A**–**D**), and the second case recovered at 9 months after surgery (**E**–**H**). At 1 month, the ELM became intact, but the ONL and EZ were disrupted in the foveal area (**B**,**F**). At 3 months, the ONL recovered its continuity but the EZ was still disconnected (**C**,**G**). After the recovery of the ELM and ONL, the continuity and hyperreflectivity of the EZ were restored.

Twenty-two cases showed an intact ELM at 1 month, 24 cases at 3 months, and 26 cases at 6 months after surgery (Fig. 2). There was no case of improvement in ELM after 6 months. The average duration of ELM recovery was 1.5 months after surgery. ONL became intact in 16 cases at 1 month, 22 cases at 3 months, and 25 cases at 6 months after surgery. No cases showed ONL improvement further since 6 months after surgery, and the average duration of ONL recovery was 2.1 months. One case at 1 month, 8 cases at 3 months, 15 cases at 6 months, 20 cases at 9 months, and 22 cases at 12 months after surgery showed intact EZ at each visit. The average duration of EZ recovery was 6.1 months. No cases showed any changes to interrupted ELM, ONL, or EZ after their recovery. It is noteworthy that all cases showing an intact ONL had an intact ELM at each follow-up visit, and cases with an intact EZ showed an intact ONL (Fig. 3).

In univariate analyses, axial length (P < 0.001), follow-up duration after surgery (P < 0.001), MLD (P = 0.001), base diameter (P = 0.029), SFT (P < 0.001), IRL thickness (P < 0.001), and PRL thickness (P = 0.031) were significant factors associated wih EZ recovery (Table 5). In multivariate analyses of model 1, axial length (P < 0.001), follow-up duration (P < 0.001), MLD (P = 0.020), and IRL thickness (P = 0.001) showed significant results. In analyses of model 2, the significant results were similar to those of model 1, with SFT also identified as a significant factor (P < 0.001).

**Table 5:** Univariate and multivariate generalized estimating equation to identify factors associated with the recovery of ellipsoid zone.

	Univariate		Multivariate (model 1)		Multivariate (model 2)	
	B (95% CI)	P value	B (95% CI)	P value	B (95% CI)	P value
Age	0.023 (− 0.008–0.053)	0.142				
Sex	− 0.583 (− 1.254 to 0.089)	0.089				
Diabetes	0.190 (− 0.704–1.084)	0.677				
Hypertension	0.241 (− 0.463–0.945)	0.502				
Axial length	− 0.001 (− 0.002–− 0.001)	** < 0.001**	− 0.001 (− 0.002 to − 0.001)	** < 0.001**	− 0.001 (− 0.002 to − 0.001)	** < 0.001**
Combined surgery	− 0.237 (− 0.899–0.425)	0.483				
F/U duration after surgery	0.326 (0.228 to 0.424)	** < 0.001**	0.460 (0.315 to 0.0.604)	** < 0.001**	0.459 (0.327 to 0.591)	** < 0.001**
Stage of MH	− 0.067 (− 0.607–0.472)	0.807				
MLD	− 0.004 (− 0.006–− 0.002)	**0.001**	− 0.007 (− 0.012–− 0.001)	**0.020**	− 0.007 (− 0.012–− 0.003)	**0.003**
Base diameter	− 0.002 (− 0.004–− 0.001)	**0.029**	− 0.002 (− 0.006–0.001)	0.185	− 0.003 (− 0.007–0.001)	0.164
Subfoveal thickness	− 0.007 (− 0.009–− 0.005)	** < 0.001**			− 0.023 (− 0.036–− 0.011)	** < 0.001**
IRL thickness	− 0.009 (− 0.011–− 0.006)	** < 0.001**	− 0.029 (− 0.045–− 0.012)	**0.001**		
ORL thickness	0.008 (− 0.006–0.021)	0.261				
PRL thickness	0.065 (0.006–0.125)	**0.031**	0.032 (− 0.098–0.161)	0.660	0.050 (− 0.077–0.177)	0.425

Values in boldface are statistically significant (P < 0.050).

*F/U* follow-up, *MH* macular hole, *MLD* minimum linear diameter, *IRL* inner retinal layer, *ORL* outer retinal layer, *PRL* photoreceptor layer.

## Discussion

We identified that the IRL tends to become thinner after FTMH surgery but still remains thicker than that of the fellow eye at 12 months after surgery. In the outer retinal layer, ELM became intact first, followed by an ONL recovery. After the recovery of the ELM and ONL, the EZ restored its continuity and hyperreflectivity. Besides recovery of the EZ, baseline BCVA, follow-up duration after surgery, MLD, IRL thickness, and SFT were significant factors affecting the change in BCVA after surgery. Additionally, axial length, follow-up duration, MLD, IRL thickness, and SFT were significant factors associated with EZ recovery.

Faria et al.^7^ reported that thinning of the GCL and IPL complex on both nasal and temporal sides of the fovea may be the major damage after FTMH surgery with ILM peel; they hypothesized that such thinning is a result of local inflammation, microcirculatory ischemia, and stretching effects by the ILM peel. Hashimoto et al.^13^ also reported that the IRL thickness progressively decreased at 6 months after surgery. Although our study showed similar trends as those of previous studies with progressive reduction of the IRL, it is noteworthy that the final thickness of the subfoveal IRL in treated eyes was thicker than in fellow eyes. The proliferation of glial cells such as Muller cells and astrocytes is known as the most important mechanism in tissue repair^14,15^. The cytoplasm of Muller cells, having a nucleus in the INL, was described as containing microfilaments, smooth endoplasmic reticulum, glycogen particles, and forming villous processes when exposed to the vitreous cavity, which may cause thickening of the IRL including the INL^14^. Such proliferation would stop after the hole is sealed. Then the organelles of Muller cells may shrink to some extent, causing a reduction of the IRL. However, irreversible structural destruction of INL by cystic lesions, localized in the INL at the edge of the FTMH preoperatively, would make the INL keep thicker than the fellow eye after surgery^16^. Further histologic studies are needed to confirm this possibility.

The PRL thickness showed a significant increase over time after surgery, and it was a significant factor associated with changes in the BCVA and recovery of the EZ in univariate analyses. Hashimoto et al.^13^ reported a significant increase in photoreceptor outer segment length and demonstrated that it was correlated with postoperative visual acuity. The increase of the PRL thickness may represent the recovery of disrupted foveal photoreceptors, causing the impact of postoperative visual acuity. However, it did not show a significant result associated with changes in the BCVA and recovery of the EZ in multivariate analyses. Although the increase of PRL thickness may mean to some extent the recovery of the photoreceptors, the recovery of the continuity and hyperreflectivity of the EZ could be also a significant factor for recovery of the photoreceptor, which cannot be demonstrated solely by the PRL thickness. Therefore, the observation of the recovery of EZ, which means continuous and hyperreflective EZ, would be more important than changes in the PRL thickness.

Gupta et al.^17^ reported that preoperative visual acuity was one of the significant predictors of visual success after macular hole surgery. Meng et al.^18^ also reported that the final BCVA was affected by the preoperative BCVA in eyes with closed macular holes. Our study showed trends similar to those of previous studies that baseline BCVA was significantly associated with changes in the BCVA after FTMH surgery. Additionally, in Table 2 showing changes in visual acuity after surgery, patients with worse baseline BCVA had limitations in improving their visual acuity compared to patients with relatively better baseline BCVA. After all, baseline BCVA could be an important predictive factor for the visual outcome of FTMH surgery. The exact mechanism is not known yet but the preoperative visual acuity may be linked to the function of the adjacent retina around the hole. The functional recovery would be better during the process of closing the macular hole by proliferative glial cells, with reapproximation of the photoreceptor layer in the retina with relatively less functional damage^14,15^.

The MLD of the FTMH was a significant factor affecting changes in the BCVA after surgery and it also showed significant results associated with the recovery of the EZ. Previous studies have reported the significance of FTMH size in surgical outcomes. Ch’ng et al.^1^ insisted that macular holes with an MLD of 650 μm could be used as a marker to predict when macular holes are more likely to close with standard surgery. Gupta et al.^17^ reported that hole size was a significant predictor of visual success after FTMH surgery. Taken together, the MLD could be a significant preoperative factor affecting anatomic and functional outcomes. In the process of sequential recovery of the ELM, ONL, and EZ after the reapproximation between the retinas at the shortest distance, the shorter the distance, the faster and better the recovery due to the lower need of horizontal stretching force to reapproximate. Whereas, the base diameter of the FTMH was not a significant factor associated with changes in BCVA and EZ recovery. It is considered that relatively fast and easy retinal reapproximation through the shorter MLD is more important for better visual outcomes than the short disconnected distance of the photoreceptor layer.

Bottoni et al.^10^ reported that the ELM is the first structure to recover after FTMH closure, and an intact ONL seems to be necessary to achieve a complete restoration of the photoreceptor microstructure. Although they predicted the earlier recovery of ONL before EZ restoration, they did not explain the time for ONL to recover; thus, the analysis of the timing of ONL recovery was insufficient. In our study, the average durations of ELM, ONL, and EZ recovery after surgery were 1.5, 2.1, and 6.1 months, respectively. All subjects with an intact EZ showed an intact ONL, and cases with an intact ONL showed an intact ELM. Thus, it seems appropriate that the order of recovery is ELM, ONL, and EZ. As such, ELM recovery may be a prerequisite for ONL recovery, and ONL recovery may be a prerequisite for EZ recovery. However, recovery of the ELM and ONL did not affect the changes in BCVA over time, unlike the results of previous studies^10,19,20^. The differences resulted from the statistical methods used in the previous studies that analyzed factors associated with the final BCVA; in contrasts, we analyzed the factors associated with changes in BCVA over time using longitudinal data. After all, the recovery of the ELM and ONL appears to be a precondition for EZ recovery, and an intact EZ could play the most important role in visual acuity improvement.

The IRL thickness was a significant factor affecting EZ recovery, which could be related to the result that the IRL thickness was also significantly associated with the changes in BCVA. The IRL contains superficial and deep capillary plexi and these support the vascular supply of the ORL including the EZ. Kim et al.^21^ reported that correlations between BCVA and the foveal avascular zone area in both superficial and deep capillary plexi were significant at 6 months after FTMH surgery. Thus, the foveal microvasculature may be significantly related to the recovery of the EZ after surgery, resulting in an improvement in BCVA. However, vitrectomized eyes for FTMH showed lower parafoveal vessel density even with a thicker IRL in the previous study^21^. IRL thickness may not represent the status of macular microvasculature directly after FTMH surgery because of the proliferation of glial cells in the IRL. Actually, the reduction in IRL was associated with BCVA improvement in our study. Such a reduction may be one of the healing processes after sealing of the FTMH, similar to the process after surgery for the epiretinal membrane, as opposed to contributing to microvasculature impairment^11,22^. Further longitudinal studies for changes in macular microvasculature using optical coherence tomography angiography are needed.

The rate of FTMH closure is known to be lower in highly myopic eyes than in normal eyes^23,24^. Additionally, Wu et al.^24^ reported that in pseudophakic eyes, the preoperative visual acuity did not differ significantly between the 2 groups, but the final visual acuity after surgery was worse in highly myopic eyes than that in control eyes. Besides the abnormality of macular structure such as posterior staphyloma, a long axial length may impair EZ recovery, as observed in the current study, resulting in lower functional visual outcomes. In addition to the thinner choroid, highly myopic eyes also have a lower microvascular density in superficial and deep retinal layers^25^. Thus, both inner and outer insufficiencies of the blood supply to the outer retina may impair EZ recovery. Further study is required to identify the exact mechanism.

Our study had several limitations. First, we did not evaluate the various visual function such as visual field tests using microperimetry after FTMH surgery. The study to analyze the change of visual function using perimetric test according to the IRL reduction is needed in the future. Second, there may have been fine segmentation errors in analyzing the retinal thickness, although we performed manual adjustments and excluded images with a definite segmentation error. Third, we did not evaluate the serial changes in the macular microvasculature after FTMH surgery, which would be meaningful using OCTA in further studies. The strength of our study is that we investigated the healing process of each retinal layer via regular follow-ups and identified factors affecting changes in BCVA and EZ recovery after FTMH surgery, which has not been reported as far as we know.

In conclusion, the IRL thickness gradually became thinner during the recovery process after the FTMH surgery but was still thicker than that of the fellow eye at 12 months after surgery. The ONL recovery occurred after the ELM became intact, and the recovery of ELM and ONL could be prerequisites for the restoration of the EZ. The BCVA change after surgery was affected by the baseline BCVA, follow-up duration after surgery, MLD, recovery of the EZ, and IRL thickness. Additionally, axial length, follow-up duration, MLD, and IRL thickness were significantly associated with EZ recovery. These findings are thought to be useful for surgeons to follow up FTMH patients postoperatively, and surgeons should carefully observe the changes in the retina including the IRL and EZ with referring above factors.
